# A Multilevel Modeling Strategy Using GULP–ORCA Integration for Crystalline Materials

**DOI:** 10.1021/acs.jpca.6c03901

**Published:** 2026-08-10

**Authors:** Anderson L. de S. Santos, Eduily B. V. Freire, Giordano F. da C. Bispo, Davi H. da S. Pedroso, Zélia S. Macedo, Mário E. G. Valerio, Gardenia S. Pinheiro, Cleânio da L. Lima

**Affiliations:** † Postgraduate Program in Physics, Campus Ministro Petrônio Portella, Federal University of Piauí, 64049-550 Teresina, Piauí, Brazil; ‡ Natural Sciences/Biology, Codó Science Center, Federal University of Maranhão, 65400-000 Codó, Maranhão, Brazil; § Brazilian Center for Research in Energy and Materials (CNPEM), Brazilian Synchrotron Light Laboratory (LNLS), 13083-970 Campinas, São Paulo, Brazil; ∥ Laboratory of Preparation and Characterization of Materials, Physics Department, 74391 Federal University of Sergipe, 49100-000 São Cristovão, Sergipe, Brazil; ⊥ Department of Physics, Campus Ministro Petrônio Portella, Federal University of Piauí, 64049-550 Teresina, Piauí, Brazil

## Abstract

Computational modeling of materials is a fundamental technological tool for scientific research. Its application enables the prediction of various material properties and provides explanations for the observed phenomena. Nevertheless, despite the range of existing computational techniques, their investigation can be challenging, especially given available computational resources. This work presents a multilevel computational methodology that integrates classical atomistic simulations performed with the General Utility Lattice Program (GULP) and electronic-structure calculations performed with ORCA software, aiming to significantly reduce the computational cost of studying crystalline materials. In this context, this study proposes a multilevel methodology for the computational investigation of crystalline materials, aiming to combine the strengths of the classical static atomistic method (CM) with DFT. The material used as the basis for the calculations was Y_2_O_3_, which is well-studied and has its properties thoroughly documented in the literature. In this multilevel methodology, optimization of the crystalline structure of Y_2_O_3_ was performed using CM, showing >99.6% agreement with experimentally measured lattice parameters and achieving a reduction of over 99% in optimization computation time compared to the DFT. Based on the structural information obtained through CM, calculations of the electronic properties of Y_2_O_3_ were performed using DFT. For this purpose, 26 different molecular structures of Y_2_O_3_ were considered with the number of atoms ranging from 5 to 350. The calculated bandgap energy values for these molecules ranged from 5.12 to 6.62 eV, and the electronic density of states (DOS) was also generated and analyzed. Calculations were additionally performed for the Y_2_O_3_ system doped with Eu^3+^ ions, where the formation of new electronic states in the DOS, attributed to the presence of the dopant, was observed.

## Graphical abstract

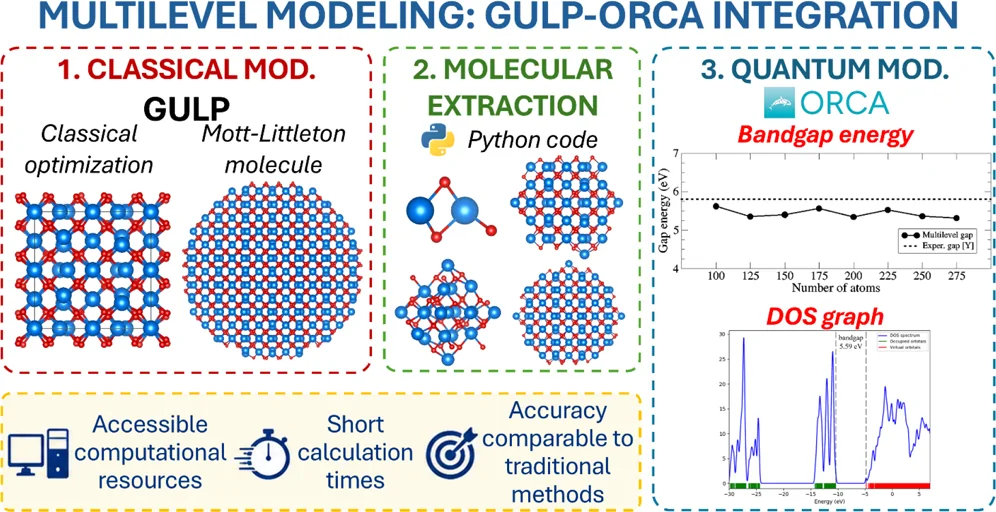

## 1 Introduction

Research in materials physics is essential for understanding a wide range of material properties and for developing innovative solutions for technological applications that enhance the quality of human life. From designing more efficient materials to discovering entirely new ones, crystalline materials attract scientific and technological interest because of their high application potential, arising from key properties such as periodic crystal structure and electrical, thermal, and mechanical behavior, among others.^1^

The complexity of crystalline structures and the variety of atomic interactions make atomic-level experimental analysis challenging for fully elucidating the properties and behavior of these materials.^2–4^ In this context, alongside various experimental techniques and the continuous progress of technology, computational materials modeling has become an essential tool in the field. Examples include density functional theory (DFT), which applies quantum mechanical (QM) principles to compute electronic and structural properties;^5,6^ molecular dynamics, which relies on atomistic interactions to simulate thermodynamic and kinetic behavior over different time scales;^7^ and classical static methods (CM), which assess equilibrium configurations and potential energy without accounting for atomic motion over time.^5,6^ Today, these approaches are essential tools that complement and strengthen experimental investigations, serving as key resources for achieving a deeper and more efficient understanding of materials.

Nevertheless, all these methodologies present complexities and challenges that must be considered in modeling work. Several reviews and research contributions have systematically addressed the inherent limitations of existing modeling strategies while clarifying the extent to which each approach matches the specific requirements of the problem at hand.^8–10^ For luminescent materials with a wide bandgap, two methodologies have been used to provide insights into the configuration of metastable levels inside the bandgap: DFT and CM modeling.^10–12^ Both provide information about lattice defects that are responsible for metastable levels.

QM methods are well-suited for describing the electronic structure of molecules and solids. For example, DFT is extensively employed in materials physics due to its versatility and robustness in studying electronic properties of systems ranging from small molecules to solids, with relatively high accuracy.^6,13^ DFT is particularly applied to the electronic structure (primarily the ground state) of many-body systems (atoms, molecules, and solids) based on the principle that the electron density contains all the information on the many-electron wave function.^13^ Consequently, the properties of a many-electron system can be computed using functionals that link the electron density to the system’s total energy.^6,13^ Despite the theoretical strength of QM and DFT, it is important to acknowledge their limitations. Notably, the computational cost can be high, especially when studying low defect concentrations in a crystalline structure, which becomes substantial for large and complex systems.

On the other hand, CM methods, such as classical static atomistic modeling, although unable to describe electronic properties or quantum interactions, have proven highly effective for investigating the structural behavior of crystalline materials. These methods rely on classical interatomic potentials and on the minimization of the crystal-lattice energy.^14–17^ This approach can address larger and more complex systems, involving tens of thousands of atoms, while maintaining low computational expense and high efficiency. Moreover, it enables screening a wide range of possible defect configurations in solids at low computational cost.

Multilevel or multiscale methodologies based on the interplay between QM and CM have gained increasing relevance in recent years, enabling the study of complex materials systems with improved accuracy and computational efficiency.^18–20^ ChemShell is a scriptable computational chemistry environment for multiscale simulations that integrate QM and molecular mechanical (MM) methods to efficiently model complex systems.^21,22^ The platform is designed for high-performance execution on parallel architecture, enabling accurate treatment of significantly larger systems. This strategy lowers computational cost and expands the scope of applications, including enzymes, catalytic surfaces, ionic materials, and microporous solids.^23^ As an example, using a hybrid QM/MM approach with the GULP and Vienna Ab-initio Simulation Package (VASP) programs, a recent study identified nitrogen vacancies as the primary defects responsible for the optical transitions observed in aluminum nitride, with simulations showing good agreement with photoluminescence measurements.^24^ Another study developed a new shell-model potential for CeO_2_ by combining Mott–Littleton defect calculations with QM/MM simulations using GULP and VASP programs. This approach integrates multiple theoretical scales to accurately reproduce charged-defect energetics, polaron behavior, and structural properties.^25^ Notably, the significance of the multiscale approach was highlighted by the 2013 Nobel Prize in Chemistry.^26^

This study proposes a multilevel strategy for the computational investigation of crystalline materials, integrating the complementary capabilities of the GULP^27^ and ORCA quantum chemistry software package.^28,29^ Rather than running both techniques in parallel, we followed a sequential workflow: the CM calculations were first performed in GULP, and their outputs were then used directly as inputs for the subsequent QM calculations in ORCA. The approach leverages the efficiency of CM modeling performed in GULP, which is well-suited for large-scale lattice simulations and defect energetics, together with the higher accuracy of DFT calculations carried out in ORCA.

The Eu-doped yttrium oxide (Y_2_O_3_) system was chosen to apply the multilevel methodology since its luminescence properties are well documented in the literature. This material presents a cubic structure with space group *Ia*3̅ and cell parameters *a* = *b* = *c* = 10.597 Å.^30^ Two nonequivalent Y sites form octahedral polyhedra, with one exhibiting centrosymmetry, whereas the other is noncentrosymmetric. Due to the chemical similarity between Y^3+^ and other trivalent rare-earth dopants, Y_2_O_3_ is known to be an excellent host for lanthanide doping. Moreover, it exhibits desirable properties such as refractory nature, stability, optical transparency over a broad spectral range, and high thermal conductivity.^31,32^ Y_2_O_3_:Eu^3+^ nanophosphors with a pure cubic phase exhibit intense red luminescence, with intensity increasing with treatment temperature, making them promising for optical applications in displays and lighting.^33^ When codoped with Tb^3+^, they demonstrate efficient energy transfer between ions, with light emission optimized at 3 mol %, highlighting their potential for electroluminescent devices.^34^ Sol–gel-synthesized Y_2_O_3_ nanoparticles doped with Er^3+^/Yb^3+^ exhibit a spherical shape, a stable cubic phase, and excellent dispersion in solvents, making them promising for thin films in photovoltaic applications.^35^ Furthermore, Y_2_O_3_ nanodisks doped with Er^3+^/Yb^3+^ exhibit green/red emission under 980 nm excitation and high thermal sensitivity.^36^ Research on point defects in the crystalline structure of Y_2_O_3_ is extensive, including investigations into the stability of different defect types,^37^ the formation of clusters,^38^ and their impact on optical properties.^39,40^ Accordingly, the established knowledge of the properties of Y_2_O_3_ and its relevance to diverse technological applications substantiates its selection as the initial subject of the proposed methodology. The crystalline structure, electronic, and thermodynamic properties of this material are well established in the literature, making Y_2_O_3_ an ideal starting point for validating the proposed methodology.

The main objective of this proposal is to integrate the strengths of different computational materials modeling strategies through a multilevel methodology, thereby offering additional capabilities in materials research while reducing the overall costs. The ability to simulate complex materials with diverse technological applications on low-cost, widely accessible computers could have a significant impact on the field of materials science.

## 2 Methodology

This study employed a multilevel approach for the computational modeling of Y_2_O_3_, combining static CM modeling and DFT in a sequential CM–QM procedure. The first stage involved CM for optimizing the crystalline structure of Y_2_O_3_. CM static calculations were performed using the GULP code,^27^ a widely used program in materials modeling that efficiently handles large systems, enabling comprehensive exploration of the solid’s structural configuration at low computational cost. Subsequently, QM calculations were performed to determine the electronic properties of the CM-optimized structures. QM was performed using the DFT method, as implemented in the ORCA program,^28,29^ with the hybrid PBE functional, which has proven effective in describing the electronic properties of the studied system.^41^ An important advantage of employing this sequential strategy is the significant reduction in computational cost while maintaining the reliability of the results.

### 2.1 Classic Modeling

The Coulomb and Buckingham interatomic potentials were employed to simulate the interactions between ion pairs in Y_2_O_3_, given its highly ionic nature. In addition, the effect of the ions’ electronic polarizability was incorporated through the Dick–Overhauser shell model.^42^ Eqs 1 and 2 represent the expressions describing the interatomic potentials and ionic polarizability, respectively.

1 $$U_{ij}=\frac{q_{i}q_{j}}{r_{ij}}+A_{ij}\exp\left(-\frac{r_{ij}}{\rho_{ij}}\right)-\frac{C_{ij}}{r_{ij}^{6}}$$

2 $$\alpha =\frac{q_{s}^{2}}{k_{cs}}$$

In eq 1, *q*_*i*_ and *q*_*j*_ are the ionic charges, *r*_*ij*_ is the distance between the ions, and *A*_*ij*_, ρ_*ij*_, and *C*_*ij*_ are the potential parameters. In eq 2, *q*_s_ represents the ion-shell charge (valence electrons), and *k*_cs_ is the coupling force constant between the ion core and shell, as described by the shell model. The parameter values used for CM static modeling of the Y_2_O_3_ crystalline structure in this study, as well as the description of interatomic interactions, follow those reported in ref 14.

In the determination of interaction potentials, the lattice-energy minimization U(r⃗) is performed by calculating the interaction energy among all *N* ions, each moving along a direction and distance proportional to the average force acting upon them. The process seeks a configuration in which the energy derivative vanishes or becomes negligible (∂*U*/∂*r⃗* ≅0), with *r⃗* being the three-dimensional position vector. The Newton–Raphson method is employed to perform this minimization,^6^ updating the ion position iteratively from the previous configuration, such that

3 $${\mathrm{r⃗}}_{n+1}={\mathrm{r⃗}}_{n}-g\left(H^{-1}\right)$$

where *n* is the iteration step, ${\mathrm{r⃗}}_{n}$ is the initial or most recently calculated position of the ion, and ${\mathrm{r⃗}}_{n+1}$ is the newly calculated position. Here, *g* is the gradient vector, and *H* is the 3*N* × 3*N* Hessian matrix, which corresponds to the first and second derivatives of the potential energy U(r⃗) , respectively. The procedure is iteratively repeated until the convergence criterion of minimum energy (∂*U*/∂*r⃗* ≅0) is fulfilled.

### 2.2 Mott–Littleton Approach

Following the simulation of the material’s crystalline lattice, the Mott–Littleton approach was applied to generate a large spherical Y_2_O_3_ molecule, optimized using CM static techniques.^43^ The Mott–Littleton approach is widely employed for defect studies in solids and involves partitioning the crystalline material into two spherical regions surrounding a central point. The spherical Y_2_O_3_ molecule includes all ions located within Region I of the Mott–Littleton approach, corresponding to the area containing the ions nearest to a predefined central point within the solid. To define the center of the undoped spherical molecule, the position of a host Y^3+^ ion was adopted as a reference. For molecules containing a defect (see Figure 1(b)), the center was determined based on the precise location of the Eu^3+^ dopant. In this way, the total energy of a lattice containing a defect using the Mott–Littleton approach is given by

4 $$U_{\mathrm{def}}=U_{I}\left(\mathrm{x⃗}\right)+U_{IIa}\left(\mathrm{x⃗},{\mathrm{\zeta ⃗}}_{e}\right)-\frac{1}{2}\left[{\frac{\partial U_{IIa}\left(\mathrm{x⃗},\mathrm{\zeta ⃗}\right)}{\partial \mathrm{\zeta ⃗}}|}_{\mathrm{\zeta ⃗}={\mathrm{\zeta ⃗}}_{e}}\right]{\mathrm{\zeta ⃗}}_{e}$$

where *U*_I_ and *U*_IIa_ are the interaction energies of the ions within regions I and IIa, respectively. The vector *x⃗* represents the positions of the ions in region I. At the same time, ζ⃗ is the induced displacement vector of the ions in region IIa, with ${\mathrm{\zeta ⃗}}_{e}$ being its equilibrium value. For this study, the diameter of region I was set to 36 Å, containing 1655 ions.

**Figure 1:**
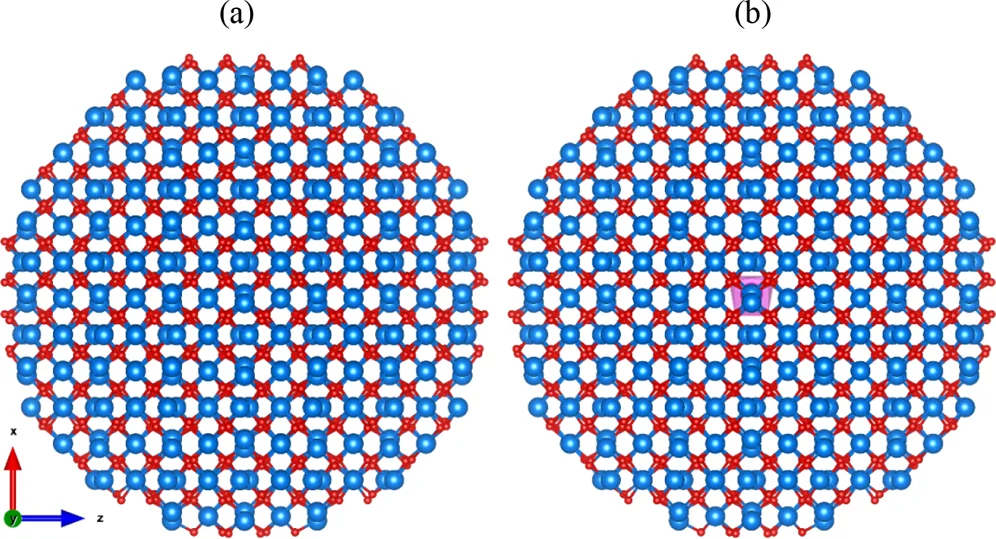
Illustration of the spherical molecular structure of Y_2_O_3_ containing 1655 ions and a radius of 36 Å, generated via CM using the Mott–Littleton approach. (a) Undoped Y_2_O_3_ molecule and (b) Eu^3+^-doped Y_2_O_3_, with the dopant located at the molecular center. In the figure, blue spheres denote Y ions, red spheres represent O ions, and pink spheres indicate Eu ions.

The incorporation of dopants into a crystal lattice may be modeled as a solid-state reaction, which simulates real crystallization mechanisms through fusion or chemical substitution during synthesis.^12,15^ For precise modeling, all reactants and byproducts must be accounted for, ensuring the conservation of ions, charge, and crystal sites. This approach enables the calculation of an accurate energy balance for each reaction using the so-called solution energy. The solid-state reaction for Eu^3+^ doping in Y_2_O_3_ is expressed as

5 $$\frac{1}{2}Eu_{2}O_{3}+Y_{Y}\to \left(Eu_{Y}\right)+\frac{1}{2}Y_{2}O_{3}$$

Figure 1(a,b) illustrates the classically optimized spherical Y_2_O_3_ molecule undoped and with a trivalent europium dopant (Eu^3+^), respectively.

All molecules analyzed in this study were derived from the spherical molecule containing 1655 atoms, which had been previously optimized using the CM methodology with the Mott–Littleton approach. To create molecular structures of varying sizes, the authors developed a computational code that constructed these structures in a specific way. The algorithm selects the atoms closest to the central point of the original spherical molecule to achieve a desired molecular size. Throughout this process, the original stoichiometric ratio of the molecular formula (Y_2_O_3_) is preserved, maintaining the relative proportions of each chemical species in the generated structures.

### 2.3 Quantum Modeling

Following the calculations and detailed determination of the energetically most favorable structural configuration of Y_2_O_3_ (classically optimized), these results were used to compute electronic properties using the DFT method. Structural optimization within DFT was disabled, and a single SCF iteration was performed. The PBE0 hybrid functional (the one-parameter hybrid version of PBE),^44^ a reliable functional that combines the so-called PBE generalized-gradient functional with a predefined amount of exact exchange, was employed according to the expression

6 $$E_{XC}=E_{DFT}^{X}+a^{\prime }\left(E_{HF}^{X}-E_{DFT}^{X}\right)+E_{DFT}^{C}$$

with *a*′ = 1/4, where *E*_XC_ is the total exchange–correlation energy, *E*_HF_^X^ is the Hartree–Fock exchange, *E*_DFT_^X^ is the DFT exchange, and *E*_DFT_^C^ is the DFT correlation energy.

The Def2-SVP (split-valence polarized) basis set was employed, which is a valence double-ζ basis set with polarization functions.^45^ These basis sets are all-electron for elements H–Kr and automatically load Stuttgart–Dresden effective core potentials for elements Rb–Rn. Therefore, they are appropriate for use with Y and Eu.

Bandgap energies were determined from the difference between the highest occupied molecular orbital (HOMO) and the lowest unoccupied molecular orbital (LUMO), i.e., *E*_g_ = |*E*_HOMO_ – *E*_LUMO_|. Additionally, the density of states (DOS) was calculated for each generated molecule.

### 2.4 Multilevel Approach

The multilevel approach employed in this study aims to combine speed, efficiency, and accuracy, serving as an essential tool for investigating and understanding the properties of complex materials at the atomic scale.

This strategy follows a sequential scheme: initially, the crystal structure is optimized through CM simulations within the GULP program using classical potentials. Subsequently, molecular structures of different sizes are generated from the classically optimized structure while maintaining stoichiometry, that is, integer multiples of the Y_2_O_3_ molecule ((Y_2_O_3_)_*n*_, com *n* = 1, 2, 3...). Finally, the electronic properties of the (Y_2_O_3_)_*n*_ molecules are calculated using the ORCA program, without requiring further structural optimization. This strategy builds on the well-established practice of using CM for structural optimization in structural and energetic studies, as many materials possess interatomic potentials that are satisfactorily parametrized.^46–49^ In such cases, the classically optimized geometry provides a realistic and stable representation of the crystalline structure, making it an appropriate starting point for the QM stage.

In this study, two desktop computers were used for performing the calculations. The CM simulations were conducted on a system equipped with an Intel Core i5-3330 processor, 8 GB of RAM, a 240 GB SSD, and integrated Intel HD Graphics 2500, running Ubuntu 22.04. The QM calculations were performed on a separate system with an Intel Core i7-13700 processor, 16 GB of RAM, a 512 GB SSD, a dedicated NVIDIA GeForce RTX 3050 graphics card, and a Windows 11 operating system.

## 3 Results and Discussion

### 3.1 Optimization of the Crystalline Structure

The interatomic potentials applied in this study are identical to those used in ref 14, which reproduced the lattice parameters of the crystalline structure with agreement better than 99.6%. Figure 2 illustrates the comparison between experimental and theoretical X-ray diffraction (XRD) data for Y_2_O_3_. Specifically, Figure 2 compares the simulated XRD patterns of Y_2_O_3_ with experimental measurements reported in ref 30, demonstrating good agreement and validating the accuracy of the theoretical predictions. Table 1 provides a detailed comparison of the lattice parameters of the experimental and calculated Y_2_O_3_ crystal structures.

**Figure 2:**
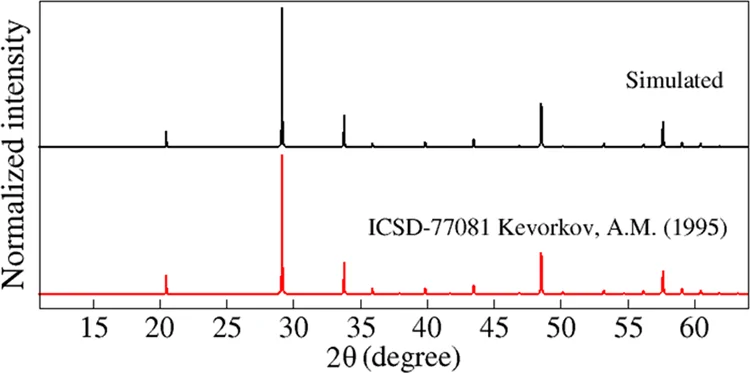
XRD pattern of Y_2_O_3_ optimized using CM. The red curve corresponds to the experimental data reported in ref 30, whereas the black curve represents the simulated XRD pattern.

**Table 1:** Comparison between Experimental and Simulated Lattice Parameters of Y_2_O_3_.

parameter	experimental^30^	calculated	Δ %	time
*a* = *b* = *c*	10.597 Å	10.556 Å	–0.38	1.03 s
α = β = γ	90.0°	90.0°	0.00	

Following structural optimization, the molecular structures of (Y_2_O_3_)_*n*_ with varying sizes were generated. A total of 27 distinct molecular structures were analyzed, of which 16 correspond to *n* values ranging from 1 to 16, and the remaining 11 to *n* values between 20 and 70 at regular intervals of 5 units. Table 2 illustrates some of the Y_2_O_3_ molecules used with Y ions in blue, O ions in red, and Eu ions in pink.

**Table 2:**
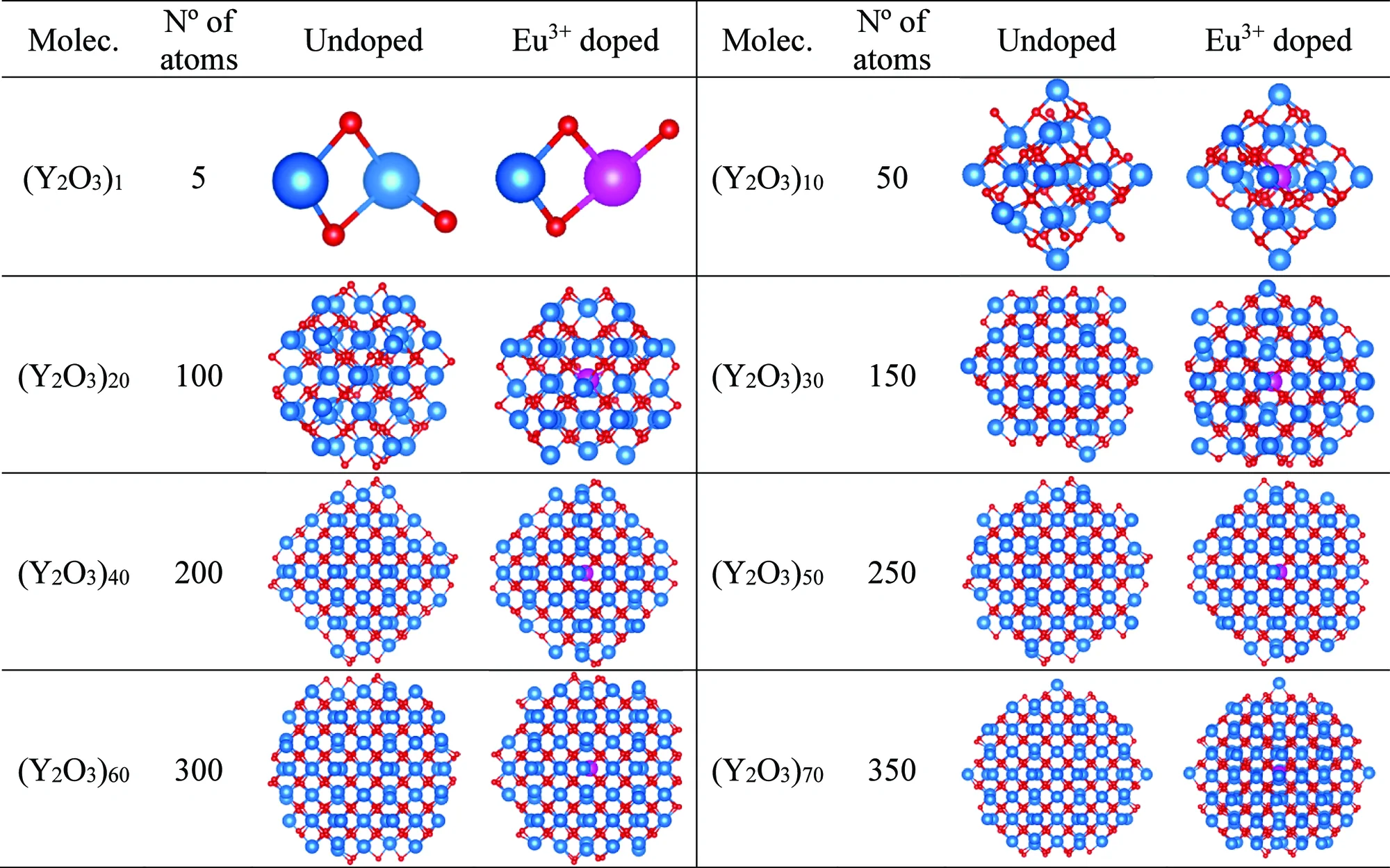
Illustration of Selected Y_2_O_3_ Molecular Structures, Both Undoped and Eu-Doped, Used for QM Calculations^a^

a Y ions are shown in blue, O ions in red, and Eu ions in pink.

### 3.2 Bandgap Energy

After disabling geometry optimization of each molecule’s crystalline structure, the HOMO–LUMO energy differences were computed for all molecules. This difference corresponds to the bandgap energy, a key property for predicting a material’s electrical conductivity. The calculated bandgap values for (Y_2_O_3_)_*n*_ molecules with *n* = 1–16 are presented in Figure 3. The figure also includes values from conventional DFT calculations reported in the literature,^50–52^ obtained using different programs and functionals, along with the bandgap value of 5.8 eV.^53–55^

**Figure 3:**
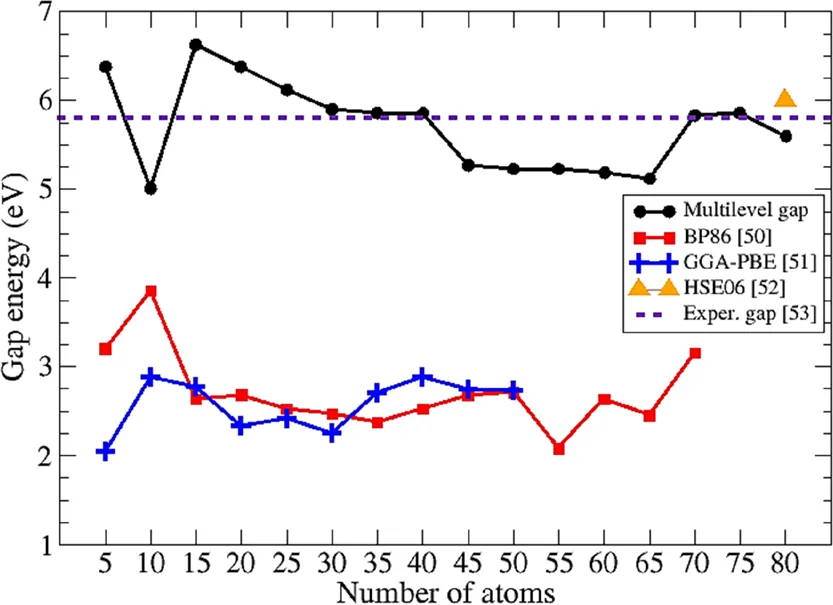
Comparison of the bandgap energies obtained using the proposed multilevel strategy with literature values. The “BP86” data were adapted with permission from ref 50 Copyright 2021 Wiley. The “GGA-PBE” data were adapted from ref 51 Copyright 2013 American Chemical Society. The “HSE06” and experimental values were taken from refs 52 and 53 , respectively.

As shown in Figure 3, the bandgap energies calculated with the proposed multilevel methodology for (Y_2_O_3_)_*n*_ (for *n* = 1–16) range from 5.00 to 6.62 eV. These results are consistent with the bandgap energy of Y_2_O_3_ reported in the literature.^53,55^ The multilevel approach was also applied to larger Y_2_O_3_ molecules than those presented in Figure 3. Figure 4 presents the calculated bandgap energy values for (Y_2_O_3_)_*n*_ molecules with *n* = 20, 25, 30, 35, 40, 45, 50, and 55, where the number of atoms varies between 100 and 275.

**Figure 4:**
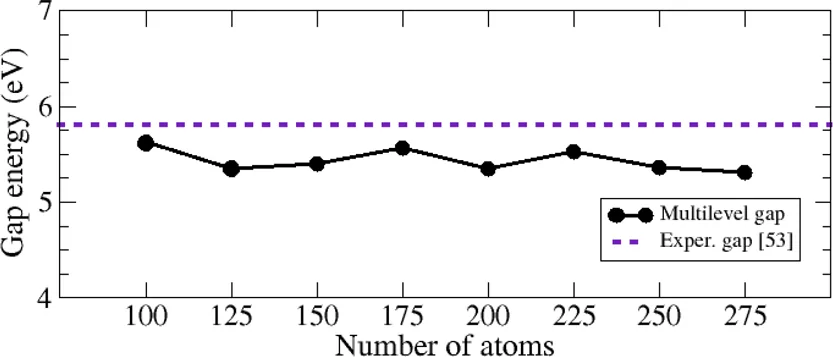
Bandgap energy of (Y_2_O_3_)_*n*_ molecules, with *n* = 20–55.

As shown in Figure 4, the bandgap energies of the larger Y_2_O_3_ molecules exhibited fewer fluctuations when compared with those of the smaller molecules illustrated in Figure 3. Such stability may be attributed to the total number of ions within the molecule because an increased number of internal ions generally mitigates the impact of edge and surface effects. Consequently, the computed results exhibit a lower variability. The bandgap values remain consistent with the experimental reference of 5.8 eV,^55^ ranging from 5.31 to 5.62 eV.

As an example, Figure 5 shows the total DOS for the (Y_2_O_3_)_16_ and (Y_2_O_3_)_35_ molecules containing 80 and 175 ions, respectively.

**Figure 5:**
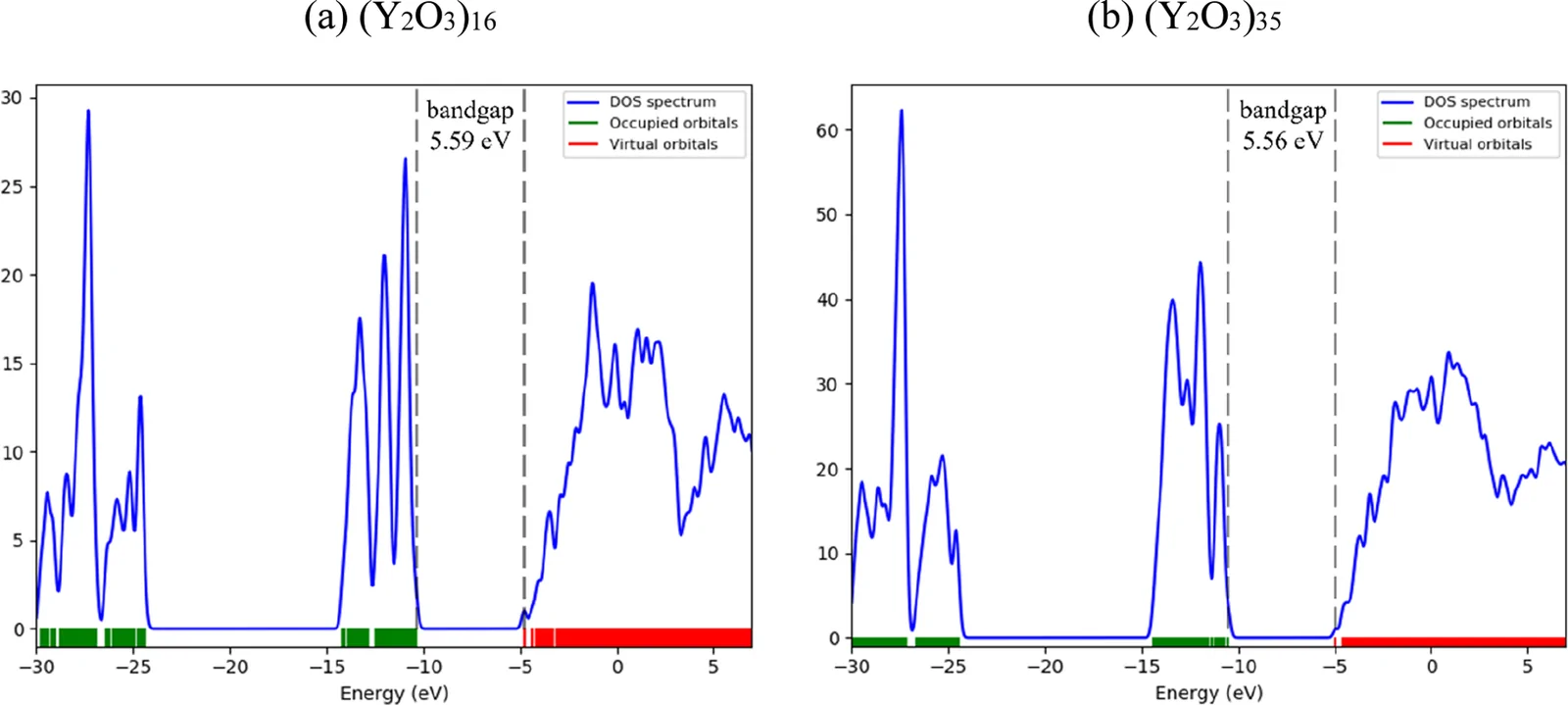
Total DOS calculated for the undoped (a) (Y_2_O_3_)_16_ and (b) (Y_2_O_3_)_35_ molecules using the multilevel methodology. The small vertical bars along the energy axis indicate the distribution of electronic orbitals. The green bars represent the occupied states in the valence band, while the red bars correspond to the unoccupied states in the conduction band.

For (Y_2_O_3_)_16_, the HOMO and LUMO were found at −10.37 eV and −4.78 eV, respectively, yielding a bandgap of 5.59 eV (Figure 5(a)). In the case of the (Y_2_O_3_)_35_ molecule, the HOMO and LUMO levels were located at −10.51 eV and −4.95 eV, respectively, corresponding to a bandgap of 5.56 eV (Figure 5(b)). These results demonstrate that both molecules, along with the other undoped structures examined (Table 2), exhibit bandgaps in good agreement with those reported in the literature (5.80 eV).^55^

### 3.3 Calculation of Eu^3+^-Doped Y_2_O_3_

Additionally, multilevel approach calculations were performed for the Y_2_O_3_ compound doped with Eu^3+^ ions, incorporated into the crystal lattice through isovalent substitution of Y^3+^ ions, as illustrated by the solid-state reaction in expression (5). Figure 6 presents the total DOS plots for the molecular structures (Y_2_O_3_)_*n*_:Eu^3+^ for *n* = 60, 65, and 70

**Figure 6:**
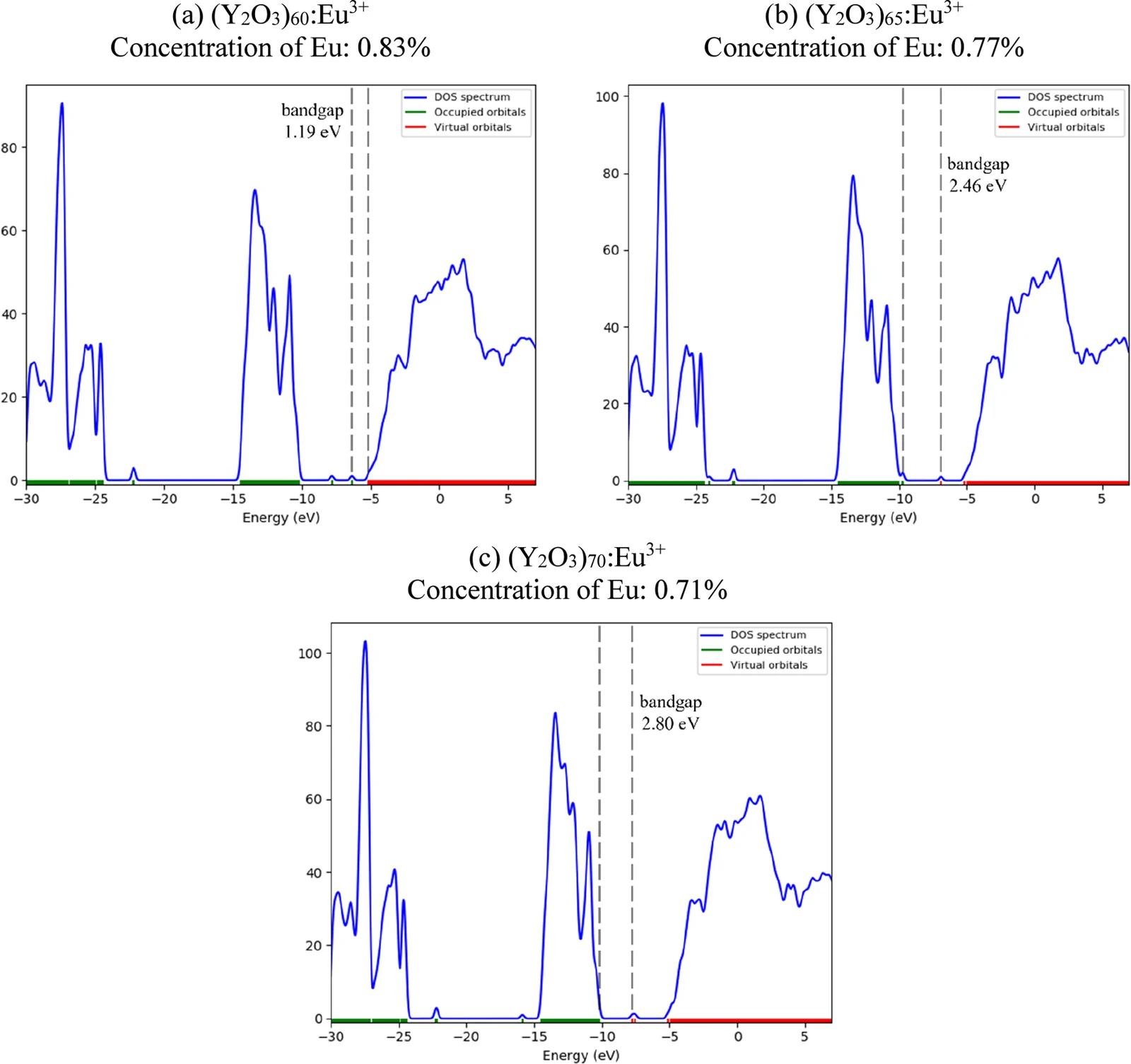
Total DOS calculated for the Eu^3+^-doped (a) (Y_2_O_3_)_60_, (b) (Y_2_O_3_)_65_, and (c) (Y_2_O_3_)_70_ molecules using the multilevel methodology. The small vertical bars along the energy axis indicate the distribution of electronic orbitals. The green bars represent the occupied states in the valence band, while the red bars correspond to the unoccupied states in the conduction band.

Experimentally, the bandgap of Y_2_O_3_:Eu^3+^ has been reported in the range of 4.5–5.25 eV, depending on the Eu^3+^ concentration (0.6–9.0%).^56–58^ However, in all three plots, the formation of new electronic states induced by the Eu^3+^ dopant is observed, leading to a modification of the host bandgap. The apparent reduction of the HOMO–LUMO difference is associated with the presence of Eu levels inside the forbidden band, which yields an apparent underestimation of the bandgap. Such behavior is characteristic of doping processes in insulating materials, where the introduction of dopant ions creates intermediate levels within the forbidden band. Consequently, the effective bandgap is reduced, promoting optical phenomena such as absorption in new spectral regions and luminescent emission associated with localized electronic states.

### 3.4 Calculation Time

The multilevel approach offers the advantage of combining the strengths of multiple computational modeling strategies, leading to reduced calculation times. Table 3 presents the computational times for molecules of different sizes assessed in this study for both Eu^3+^-doped and undoped cases.

**Table 3:** Computational Times Required for the Calculations Using the Multilevel Methodology for all Y_2_O_3_ Molecular Structures, Both Undoped and Eu^3+^-Doped, Analyzed in This Study

		undoped	Eu^3+^ doped			undoped	Eu^3+^ doped
molecule	no of atoms	time	time	molecule	no of atoms	time	time
(Y_2_O_3_)_1_	5	10 s	1 m 08 s	(Y_2_O_3_)_15_	75	1 m 40 s	2 m 19 s
(Y_2_O_3_)_2_	10	05 s	1 m 08 s	(Y_2_O_3_)_16_	80	1 m 52 s	3 m 24 s
(Y_2_O_3_)_3_	15	07 s	1 m 11 s	(Y_2_O_3_)_20_	100	3 m 07 s	3 m 58 s
(Y_2_O_3_)_4_	20	08 s	1 m 13 s	(Y_2_O_3_)_25_	125	5 m 12 s	7 m 43 s
(Y_2_O_3_)_5_	25	12s	1 m 16 s	(Y_2_O_3_)_30_	150	8 m 00 s	8 m 20s
(Y_2_O_3_)_6_	30	16 s	1 m 22 s	(Y_2_O_3_)_35_	175	10 m 23 s	13 m 14 s
(Y_2_O_3_)_7_	35	21 s	1 m 28 s	(Y_2_O_3_)_40_	200	14 m 18 s	17 m 21 s
(Y_2_O_3_)_8_	40	27 s	1 m 35 s	(Y_2_O_3_)_45_	225	18 m 32 s	23 m 16 s
(Y_2_O_3_)_9_	45	35 s	1 m 44 s	(Y_2_O_3_)_50_	250	23 m 52 s	33 m 49 s
(Y_2_O_3_)_10_	50	42 s	1 m 59 s	(Y_2_O_3_)_55_	275	29 m 27 s	36 m 12 s
(Y_2_O_3_)_11_	55	51 s	2 m 08 s	(Y_2_O_3_)_60_	300	36 m 17 s	43 m 03 s
(Y_2_O_3_)_12_	60	1 m 01 s	2 m 14 s	(Y_2_O_3_)_65_	325	39 m 58 s	52 m 05 s
(Y_2_O_3_)_13_	65	1 m 09 s	2 m 28 s	(Y_2_O_3_)_70_	350	50 m 38 s	1 h 00 m 13 s
(Y_2_O_3_)_14_	70	1 m 21 s	2 m 43 s				

It was observed that calculations for the crystalline structure of Eu^3+^-doped Y_2_O_3_ were more computationally demanding than those for pure Y_2_O_3_. This difference was anticipated, given the local effects of the dopant ion, since both electronic correlation and symmetry breaking induced by the defect strongly affect the calculations, especially for larger molecular systems. In addition, the use of the multilevel methodology led to a significant reduction in computational time compared to the conventional DFT approach. This reduction is directly associated with the fact that in the multilevel workflow, structural optimization is not performed within DFT. Because the DFT-based optimization step is highly demanding in terms of computational resources, employing CM methods for the initial optimization justifies the reduction in the computational time observed. For comparison purposes, calculations were also performed by using the conventional DFT approach on a subset of the molecules listed in Table 2. In these calculations, the same functional and basis sets used in the multilevel methodology were adopted. Table 4 summarizes the computational times required for the (Y_2_O_3_)_*n*_ molecules with n = 1–4, for both the multilevel approach and traditional DFT.

**Table 4:** Comparison of the Computational Times Required for Y_2_O_3_ Molecular Structures Calculated Using the Multilevel Methodology and Conventional DFT, Performed on the Same Computer System

	multilevel	DFT	
molecule	time	time	reduction (%)
(Y_2_O_3_)_1_	05 s	11 m 15 s	99.259
(Y_2_O_3_)_2_	06 s	1 h 00 m 30 s	99.834
(Y_2_O_3_)_3_	08 s	17 h 13 m 16 s	99.987
(Y_2_O_3_)_4_	09 s	28 h 30 m 06 s	99.991

The contrast in the computational cost between the two approaches is notable. Using the multilevel methodology, all molecules listed in Table 4 were processed in under 10 s. In comparison, conventional DFT calculations required substantially more time, taking over a day for the (Y_2_O_3_)_4_ molecule. This result highlights the high computational cost associated with the structural optimization step in conventional DFT, which entails iterative self-consistent calculations to achieve electronic and geometric convergence, resulting in long processing times.

## 4 Conclusions

This study proposes a multilevel strategy for integrating the GULP and ORCA programs, directly employing the results from each stage to support the computational investigation of crystalline materials and combining static CM modeling with DFT calculations.

The crystalline structure of Y_2_O_3_ was optimized via CM, achieving an over 99.6% agreement with the experimentally determined lattice parameters. Moreover, this approach enabled a reduction of more than 99% in optimization time relative to DFT calculations for different molecular sizes on the same computational setup.

Using the structural data obtained via CM, the electronic properties of Y_2_O_3_ were calculated by using the DFT method. For this purpose, a computational algorithm was developed to generate 26 distinct molecular structures of Y_2_O_3_ with the number of atoms varying from 5 to 350. The calculated bandgap values for these molecules ranged from 5.12 to 6.62 eV, while the value reported in the literature corresponds to 5.8 eV. For larger molecular structures, comprising between 70 and 275 atoms, the bandgap values obtained were between 5.83 and 5.31 eV. The DOS analysis further revealed the detailed distribution of the electronic orbitals in the Y_2_O_3_ molecules. Moreover, calculations performed for Y_2_O_3_ doped with Eu^3+^ ions showed the emergence of additional electronic states in the DOS spectrum, arising from the presence of the dopant, which modifies the electronic properties of the system and is relevant to optical applications.

These results demonstrate that the proposed multilevel strategy is capable of efficiently describing both structural and electronic properties at a low computational cost, even when employing simple computers readily available to most researchers. Integrating the strengths of different computational approaches to material simulation introduces a novel means of exploring and understanding materials, leading to a substantial reduction in costs. The specific integration strategy implemented between the GULP and ORCA programs offers a practical and efficient means of investigating the structural and electronic properties of materials, leading to a substantial reduction in the computational cost. Moreover, the ability to address more complex materials with diverse technological applications by using widely accessible computers could have a positive impact on materials research.
